# The feasibility and utility of hair follicle sampling to measure FMRP and *FMR1* mRNA in children with or without fragile X syndrome: a pilot study

**DOI:** 10.1186/s11689-022-09465-7

**Published:** 2022-12-09

**Authors:** Isha Jalnapurkar, Jean A. Frazier, Mark Roth, David M. Cochran, Ann Foley, Taylor Merk, Lauren Venuti, Lucienne Ronco, Shane Raines, Diego Cadavid

**Affiliations:** 1 Department of Psychiatry, EK Shriver Center, University of Massachusetts Medical School, Worcester, MA USA; 2 Fulcrum Therapeutics, Cambridge, MA USA

**Keywords:** Fragile X, *FMR1* mRNA, FMRP, Hair follicle, Clinical biomarker

## Abstract

**Background:**

Fragile X syndrome (FXS) is the most common inherited cause of intellectual disability in males and the most common single gene cause of autism. This X-linked disorder is caused by an expansion of a trinucleotide CGG repeat (> 200 base pairs) on the promotor region of the *fragile X messenger ribonucleoprotein 1 gene (FMR1)*. This leads to the deficiency or absence of the encoded protein, fragile X messenger ribonucleoprotein 1 (FMRP). FMRP has a central role in the translation of mRNAs involved in synaptic connections and plasticity. Recent studies have demonstrated the benefit of therapeutics focused on reactivation of the *FMR1* locus towards improving key clinical phenotypes via restoration of FMRP and ultimately disease modification. A key step in future studies directed towards this effort is the establishment of proof of concept (POC) for FMRP reactivation in individuals with FXS. For this, it is key to determine the feasibility of repeated collection of tissues or fluids to measure *FMR1* mRNA and FMRP.

**Methods:**

Individuals, ages 3 to 22 years of age, with FXS and those who were typically developing participated in this single-site pilot clinical biomarker study. The repeated collection of hair follicles was compared with the collection of blood and buccal swabs for detection of *FMR1* mRNA and FMRP and related molecules.

**Results:**

There were *n* = 15 participants, of whom 10 had a diagnosis of FXS (7.0 ± 3.56 years) and 5 were typically developing (8.2 ± 2.77 years). Absolute levels of FMRP and *FMR1* mRNA were substantially higher in healthy participants compared to full mutation and mosaic FXS participants and lowest in the FXS boys. Measurement of *FMR1* mRNA and FMRP levels by any method did not show any notable variation by collection location at home versus office across the various sample collection methodologies of hair follicle, blood sample, and buccal swab.

**Conclusion:**

Findings demonstrated that repeated sampling of hair follicles in individuals with FXS, in both, home, and office settings, is feasible, repeatable, and can be used for measurement of *FMR1* mRNA and FMRP in longitudinal studies.

**Supplementary Information:**

The online version contains supplementary material available at 10.1186/s11689-022-09465-7.

## Introduction

Fragile X syndrome (FXS) is an X-linked genetic condition associated with an expansion of the trinucleotide (cytosine-guanine-guanine) CGG repeat within the 5′-untranslated region of the *fragile X messenger ribonucleoprotein 1* (*FMR1*) gene [1]. The majority of cases are caused by epigenetic silencing resulting from expanded CGG repeats in exon 1 of the *FMR1* gene on the X chromosome that results in hypermethylation of the promoter, heterochromatin formation, and prevention of gene transcription. This results in a deficit of the *FMR1*-encoded protein fragile X messenger ribonucleoprotein 1 (FMRP), an RNA-binding protein that regulates dendritic translation and plays a critical role in synaptic development and function [2] FXS can also be caused by mosaicism of transcriptional silencing of the gene, occurring in some but not all of the cells due to either varying size of the repeat expansion or variations in methylation patterns. Mosaicism can result in variability in the production of FMRP [3–6].

FXS is the most common inherited cause of intellectual disability with a prevalence of 1 in 4000–7000 for males and 1 in 8000–11,000 for females [7]. Boys are generally more severely affected because the presence in all girls of a second healthy X chromosome with random inactivation of either the healthy or mutated X chromosome in each cell determines the clinical phenotype [8]. Variability in methylation and instability of the repeat expansion also contribute to phenotypic heterogeneity. FMRP is expressed in various tissues in the central nervous system (CNS) and periphery and is responsible for several functions including neurogenesis, synaptic plasticity, ovarian functions, and neuropsychiatric symptoms [9]. Clinical manifestations are diverse and vary from mild to severe intellectual disability with variable behavioral impairments which may be related to the level of FMRP [4, 5, 10, 11]. The most notable clinical phenotypes are deficits in expressive language development and impairment of social interactions with one or more of the following: social anxiety, hyperactivity, and sensory hypersensitivity [5, 12]. Autism spectrum disorder (ASD) is a frequent comorbid condition seen in 30 to 43% of males with FXS and 16 to 20% of females with FXS [13]. FXS is the most common known single-gene cause of ASD [14–17].

This wide array of cognitive, emotional, and systemic challenges can have significant effects on the academic and daily functioning of individuals with FXS [13, 18]. The majority of the current symptom-based pharmacological treatments are based on FDA-approved treatments for these conditions in the general population or those with other neurodevelopmental disorders [13, 19]. Advances in experimental models of FXS and other neurodevelopmental disorders with known genetic origins have paved the way for the potential development of disease- and neurobiologic mechanism-specific pharmacological treatments [20–22]. These include novel treatments that target the core deficits at the cellular level, including immature synaptic connections, altered synaptic plasticity, and impaired memory formation, which occur due to lack of FMRP [21–23].

An innovative approach to therapeutic development in FXS involves directly targeting the proximal event in disease pathogenesis—the transcriptional silencing of the *FMR1* gene [24]. A limited number of studies utilizing pharmacological approaches to reactivate the *FMR1* locus have met with success [25, 26]. An important step in enabling testing of potential treatments for FMR1 reactivation is development of translational methods to measure FMR1 mRNA and FMRP in early, proof-of-concept (POC) studies. Therefore, it is critical to be able to collect tissues and/or fluids that can be sampled repeatedly and safety and used to measure changes in mRNA and protein. Traditionally, this has been done with PBMCs. However, scalp hair follicles have several potential advantages to study treatment candidates for FMR1 reactivation in people affected by FXS: (1) they are amenable to repeated sampling with little pain and discomfort; (2) they self renew; (3) hair follicles, similar to neurons, develop from cells in the cranial ectoderm, which is the precursor of the central nervous system and skin and may be subject to similar epigenetic silencing early in development; and (4) FMRP expression in hair follicles appears to be clonal in origin, as it is absent in all follicles in non mosaic males while absent only in about half of follicles from females with the FMR1 repeat expansion mutation, consistent with random chromosome X inactivation [27]. Previously, Willemsen et al. [27] used antibody detection of FMRP in hair follicles for successful diagnosis of FXS. In his pioneer studies, Willemsen et al. [27] showed that hair follicle removal by plucking is feasible and well-tolerated in children with FXS, and that FMRP is readily detectable by immunohistochemistry (IHC) in hair follicles of unaffected relatives and less affected patients. He reported that the presence of FMRP in hair follicles by IHC was more predictive of intellectual disability as measured by IQ testing than FMRP detection in blood. Several key proteins involved in cellular differentiation and signal transduction, neurotrophin receptors, and cell adhesion molecules are shared between neurons and hair follicle cells, in addition to FMRP [28]. In a recent study, we found a 0.59 correlation between FMRP measured in isolated peripheral blood mononuclear cells (PBMCs) and intellectual disability in males ages 3–74 years ranging from normal to full mutation [5]. However, there is little experience with the feasibility of serial collection of hair follicles by plucking in the clinic as the work by Willemsen et al. was done at home. Additionally, optimal (sensitive, specific, and reliable) methods for the measurement of *FMR1* mRNA, FMRP, and related molecules in human tissues are only recently being developed by us [5] and others [6]. In addition to the identification of a biosample source for repeated sampling of tissue during clinical trials, it is crucial to incorporate appropriate outcome measures to evaluate future clinical therapeutic trials. Thus, a major goal of this manuscript is the measurement of FMRP and *FMR1* mRNA in hair follicles in order to achieve more CNS-relevant measurements and, consequently, more meaningful FMRP/*FMR1 mRNA*-clinical correlations.

The following questions were investigated by this pilot study:

1. Is repeated sampling of hair follicles by plucking feasible in children with or without fragile X syndrome at home and in the clinic?
2. Is the measurement of the presence and amount of FMRP by Meso Scale Discovery enzyme-linked immunosorbent assay (MSD ELISA) less variable and more reliable than with IHC?
3. Are hair follicles a more feasible choice for repeated sampling of tissue/fluid from children with FXS than blood collection from peripheral veins and/or buccal swabs?
4. Which of the fluids/tissues tested for *FMR1* mRNA and FMRP better reflect cognitive function, as measured by tests of oral expression and listening comprehension?

## Methods

This was a small, single-center (University of Massachusetts Chan Medical School (UMass Chan)), prospective, nondrug pilot feasibility study of the repeated collection of hair follicles by plucking in individuals, 3 to 22 years of age, with FXS and those who were typically developing for the quantitative measurement of *FMR1* mRNA and FMRP. The repeated collection of hair follicles was compared with the collection of blood and buccal swabs for detection of *FMR1* mRNA and FMRP and related molecules in children and young adults with FXS and age- and sex-matched children and young adults without FXS (healthy controls). For this study, most participants completed three study visits: screening visit, visit 1, and visit 2. An unscheduled visit was allowed for additional sample collection, should any of the samples be inadequate for analysis, or if there was a reason the participant could not provide one of the samples at the regularly scheduled visits. If requested by the family and deemed appropriate by the principal investigator (PI), the screening visit and visit 1 could be combined. There were two sample collection visits, one in the child’s home and one at the clinic. The order of the location of the visits could vary (home visit first, office visit second or office visit first, home visit second), depending on the preference of the family. The feasibility for the repeated collection was determined by the two separate visits, 1–59 days apart. There could be one additional unscheduled visit, either in the office or at home, in case of missed or incomplete scheduled visits.

Measurement of FMRP in hair follicles and peripheral blood lymphocytes (PBLs) was performed by MSD ELISA on whole protein extracts. For PBLs, an independent, flow cytometric method was used to simultaneously measure *FMR1* mRNA and FMRP in addition to the MSD ELISA [20].

PrimeFlow™ flow cytometric assay: For a detailed protocol, see Roth et al. (2021) [5]. PrimeFlow™ was carried out per manufacturer instructions with the addition of surface and intracellular protein staining. Surface CD markers identified were CD8a, CD19, CD14, CD3, and CD4. A fixable viability dye was also included. Samples were fixed, washed twice, and permeabilized. Anti-FMRP or mouse IgG1 isotype was added for internal staining. After incubation and washes, samples were fixed and washed again. Probes to detect FMR1 or dapB mRNA were added. Following hybridization of probes, the samples were washed and stored overnight at 4 °C. The next day, the PreAMP mix was added followed by incubation and washing. RNA AMP Mix was added. After incubation and washes, PrimeFlow™ RNA-labeled probes were added. After incubation and washes, the data was acquired on an ATTUNE NXT cytometer. Data was analyzed using FlowJo software.

Absolute quantification of FMRP by electrochemiluminescence ELISA (MSD): For a detailed protocol, see Roth et al. (2021) [5]. A participant’s lysate, 150 μg/mL FMRP-positive control lysate, 150 μg/mL FMRP negative control lysate, or a standard curve of recombinant FMRP were mixed 1:1:1 with custom biotinylated rabbit, polyclonal anti-FMRP, and mouse monoclonal 6B8/FMRP in a V-bottom, polypropylene, 96-well plate. The plate was sealed and placed on a shaker at 4 °C overnight. A total of 5 μL of the mixture was added to each well of a 384-well avidin-coated MSD plate in quadruplicate. Mixtures were incubated followed by three washes. The plate was blocked using 3% MSD Blocker A in the MSD wash buffer. The plate was washed followed by addition of sulfo-tagged anti-mouse. After incubation and washes, 2× MSD read buffer T was added. The plate was immediately acquired using the MESO SECTOR S 600 reader. Data was analyzed using MSD software. A standard curve in fmol was created from the recombinant FMRP. The fmol of FMRP for each lysate was calculated from the standard curve.

Total protein concentration: For a detailed protocol, see Roth et al. (2021) [5]. The sample lysates’ concentrations were below the detection limit of a BCA reaction. Therefore, the highly sensitive ProteinSimple Total Protein Detection Module was modified to determine lysate concentrations. A PBMC or hair follicle lysate standard was created from a large batch of isolated PBMCs or hair follicles, respectively. The protein concentrations of the standards were determined using the BCA assay. A 4-point standard lysate curve and lysates from the participants’ samples were prepared following the protocol provided with the total protein detection module. The prepared lysates were run in the 12–230 kDa separation module on the JESS. Data analysis was performed using Compass software. For each point on the standard cure, the area under the curve (AUC) for peaks at 48, 75, and 190 kDa was calculated. The AUCs for the same peaks in the sample lysates were determined. The values from the standard curve were used to determine the concentrations of the sample lysates.

Hair follicle qPCR protocol: Plucked hair follicles were immediately submerged in RNA later. Samples were processed using the TRI-Reagent protocol with homogenization using the Qiashredder and the Zymo Research Direct-zol MicroPrep kit. Quantitated mRNA was prepared using SuperScript IV for first-strand synthesis and Applied Biosystems PreAmp Master Kit. Commercially available TaqMan probes were used for the FMR1 transcript (Invitrogen cat. no. Hs00924547_m1). The fast advanced master mix (Applied Biosystems 4444557) was employed, and samples were read on the QuantStudio 7 thermocycler.

### Participants

Individuals 3 to 22 years of age were recruited for this study. Some participants were invited to participate from the FXS clinic at UMass Chan, while other participants were recruited from the greater New England area via referrals from patient organizations and other methods. A parent focus group was formed as part of recruitment efforts for education on the rationale for the study and to gain parent support. Recruitment materials prepared by UMass Chan were used to facilitate awareness and communication between interested families at the Center for Autism and Neurodevelopmental Disorders (CANDO) clinic at UMass Chan and University of Massachusetts Memorial Health Care. Informed consent was obtained from the parent/legally authorized representative (LAR) for children ages 3–17 years, by self (ages 18–22), or by proxy (parent or LAR of the individual). If they were capable, assent by the child or the adult, in case of a proxy or LAR, of their willingness to have samples of hair, blood, and buccal swabs collected was obtained.

The determination of the FXS phenotype regarding expressive language delay and/or social anxiety was performed by the principal investigator (JF) based on the history and physical examination, review of available medical records, and results of standardized testing during the screening visit. All subjects were assessed at screening with standard and age-appropriate tests of language development, social anxiety, social communication impairment, and other FXS phenotypes. As described above, given that social communication challenges are a key area of difficulty in FXS, it is imperative to include measures that can identify these deficits. The overarching aim of this proof-of-concept study is the generation of data for future clinical trials focused on reactivation of *FMR1* mRNA and FMRP. Thus, the primary endpoint for these studies would involve measures of social communication and expressive language, unlike IQ, which is a complex construct that cannot be used as a primary endpoint for pharmacotherapeutic interventions. Hence, the OWLS-II Listening Comprehension (LC) and Oral Expression (OE) portions were used to measure expressive language and LC [29]. The Gilliam Autism Rating Scale 3rd Edition (GARS-3) was used for the determination of the presence and severity of restrictive/repetitive behaviors, social interaction impairment, social communication impairment, altered emotional responses, cognitive style, and maladaptive speech by parent report [30].

### Procedures

#### Hair follicle collection and analysis

Plucking refers to the process of removing human hair by mechanically pulling hair follicles from the scalp. In this study, hair follicles were collected via plucking at visit 1, visit 2, and any unscheduled visits, using methodology and tweezers provided by the Epistem Ltd. Epistem and Fulcrum provided training to UMass Chan personnel via Webex with ongoing support as needed, including on the collection and the quality assessment of plucked hairs. Required supplies were provided for hair follicle storage. It was anticipated that about 20 hair follicles would be plucked per visit, of which about 15 would pass quality control. Any plucked hairs missing the follicle were discarded by Epistem or Fulcrum.

#### Collection and analysis of blood cells, cellular DNA, and plasma

Blood was collected via venipuncture at visit 1, visit 2, and any unscheduled visits. At visit 1, blood was collected for both *FMR1* mRNA methylation assessment and *FMR1* mRNA/FMRP measurement. The blood collection at visit 2 or any unscheduled Visit was optional for subjects with FXS.

Measurement of FMRP in hair follicles was performed at Fulcrum by MSD ELISA on whole protein extracts from individual hair follicles. Measurement of mRNA in hair follicles was performed by TaqMan® analysis [31]. The blood collection tubes were shipped to Fulcrum where peripheral blood lymphocytes, DNA, and serum isolation were performed. Claritas Genomics also performed the analysis for the *FMR1* gene mutation for mosaicism and length of the triple repeat expansion when not available from the clinical records. Blood lymphocytes and plasma were evaluated for the measurement of *FMR1* mRNA or FMRP. FACS analysis was performed at Fulcrum. For complete MSD ELISA and FACS methods, refer to Roth et al. (2021).

Measurement of FMRP by IHC was discontinued early due to fragility of youth hair follicles (Epistem Ltd., UK). Measurement of FMRP in serum using MSD ELISA was performed at Fulcrum or by NanoSomiX by isolation and characterization of exosomes. Buccal swabs were obtained using the manufacturer’s instructions (Puritan HydraFlock from Puritan Diagnostics [Guilford, ME]) at visit 1, visit 2, and any unscheduled visits. Measurement of FMRP was performed at Fulcrum by MSD ELISA of protein extracts. Measurement of *FMR1* mRNA in buccal swabs was performed at Fulcrum by TaqMan analysis, as described above. Measurement of *FMR1* mRNA by qRT-PCR was not done in PBMCs due to lack of sufficient sample to run both protein assays and qRT-PCR. We were able to obtain *FMR1* mRNA expression by PrimeFlow™ because the detection of *FMR1* mRNA and FMRP was done simultaneously on the same sample.

All hair follicles, blood tubes, and buccal swabs were shipped by the primary study site on the same day as the collection under conditions specified in the study reference manual. All samples were labeled with study identification number and fully anonymized prior to shipping. Fulcrum scientists shipped a portion of the hair follicles (about 5 per subject per visit) to Epistem for analysis by IHC.

### Statistical analysis

Data analysis was performed using statistical analysis software (SAS) Version 9.4. Descriptive statistics for continuous variables included number of subjects (n), mean, standard deviation (SD), median, minimum, and maximum. Summaries of change from baseline variables included only subjects who had both a baseline value and corresponding value at the timepoint of interest. Descriptive statistics for categorical data included frequency and percentage. Where appropriate, descriptive statistics were presented with 95% confidence intervals (CIs). Measures of spread (e.g., standard deviation) were reported to 2 degrees of precision more than the recorded data. Pearson’s correlation coefficients were used to assess relationships between *FMR1* mRNA and FMRP levels with measures of OWLS-II LC and OE portions and the GARS-3 subscales. For the OWLS-II and GARS-3, standard scores were used in analyses. Higher OWLS-II scores indicate better language comprehension; higher GARS-3 scores indicate more severe autistic behavior.

## Results

### Demographic and other baseline characteristics

Overall, 15 participants were enrolled in the study, with 10 participants in the FXS participant group and 5 participants in the healthy participants group. All enrolling participants (100%) completed the study, and none discontinued from the study. There were no screen failures.

The overall demographic and baseline characteristics of subjects in the All Enrolled Set are presented in Table 1. Educational indicators differed between the two groups. The mean (±SD) age of FXS participants was 7.0 ± 3.56 years. A majority of FXS participants (60.0%) were male versus 40.0% female. The mean age of healthy participants was 8.2 ± 2.77 years. A majority of healthy participants (60.0%) were female versus 40.0% male. The enrolled population was predominantly Caucasian (93.3%) and not Hispanic or Latino (93.3%).

**Table 1:** Demographics and Baseline Characteristics

	FXS Participant (N=10)	Healthy Participant (N=5)	Overall (N=15)
Age at consent (years)			
Mean (SD)	7.0 (3.56)	8.2 (2.77)	7.4 (3.27)
Median	6.0	9.0	7.0
Min, Max	3, 15	4, 11	3, 15
Sex			
Female, n (%)	4 (40.0%)	3 (60.0%)	7 (46.7%)
Male, n (%)	6 (60.0%)	2 (40.0%)	8 (53.3%)
Race			
Asian (Filipino, Korean, Laotian, Vietnamese, Japanese, Chinese), n (%)	1 (10.0%)	-	1 (6.7%)
Caucasian or White, n (%)	9 (90.0%)	5 (100.0%)	14 (93.3%)
Ethnicity			
Hispanic/Latino, n (%)	1 (10.0%)	-	1 (6.7%)
Not Hispanic or Latino, n (%)	9 (90.0%)	5 (100.0%)	14 (93.3%)
Total years in formal school			
Mean (SD)	3.6 (3.03)	3.6 (1.95)	3.6 (2.64)
Median	2.5	3.0	3.0
Min, Max	0, 10	1, 6	0, 10
Current school year			
Pre-school, n (%)	4 ( 40.0%)	1 (20.0%)	5 (33.3%)
Kindergarten, n (%)	1 ( 10.0%)	-	1 (6.7%)
Grade 1, n (%)	2 ( 20.0%)	-	2 (13.3%)
Grade 2, n (%)	-	1 (20.0%)	1 (6.7%)
Grade 3, n (%)	-	1 (20.0%)	1 (6.7%)
Grade 4, n (%)	1 (10.0%)	1 (20.0%)	2 (13.3%)
Grade 6, n (%)	1 (10.0%)	1 (20.0%)	2 (13.3%)
Grade 9, n (%)	1 (10.0%)	-	1 ( 6.7%)
Current Individualized Educational Program (IEP)			
No, n (%)	-	5 (100.0%)	5 ( 33.3%)
Yes, n (%)	10 (100.0%)	-	10 ( 66.7%)
Number of caregivers			
1, n (%)	2 (20.0%)	-	2 (13.3%)
2, n (%)	8 (80.0%)	5 (100.0%)	13 (86.7%)
Caregiver #1 highest education			
>2 Year College, n (%)	2 (20.0%)	-	2 (13.3%)
>4 Year College, n (%)	8 (80.0%)	5 (100.0%)	13 (86.7%)
Caregiver #2 highest education			
>2 Year College, n (%)	2 (20.0%)	1 (20.0%)	3 (20.0%)
>4 Year College, n (%)	6 (60.0%)	4 (80.0%)	10 (66.7%)
No Second Caregiver, n (%)	2 (20.0%)	-	2 (13.3%)

*Abbreviations: Min* minimum, *Max* maximum, *n* number of subjects, *SD* standard deviation Percentages are calculated as n/N*100. The number of subjects enrolled is used as N for all percentage calculations

Overall, the majority of FXS participants (70.0%) were currently attending either preschool (40.0%), kindergarten (10.0%), or grade 1 (20.0%), while 80% of healthy participants currently attended from grade 2 through grade 6. The mean total number of years in formal school was 3.6 ± 3.03 for FXS participants and 3.6 ± 1.05 for healthy participants. All FXS participants (100%) and no healthy participants (0%) reported participation in individualized educational programs.

A majority of FXS participants (80%) had 2 caregivers. A majority of FXS participants had caregivers with 4 years of college or more as their highest education level (80% for caregiver no. 1 and 60% for caregiver no. 2). All healthy participants (100%) had 2 caregivers of which all except 1 caregiver had 4 years of college or more as their highest education level.

Hair samples were collected at each visit. Blood collection for FMR1 mRNA methylation was only collected in 6 of 10 FXS participants (1/4 collected at home and 5/6 collected at office).

Baseline disease characteristics for the FXS participants are presented in Table 2. All FXS participants had their prior FXS diagnosis documented by genetic testing. Five FXS participants had mosaic positive disease. On both the OWLS-II LC and OE scales, participants demonstrated low oral receptive and expressive communication function at baseline; the mean standard scores were 82 ± 16.7 for OWLS-II LC and 73 ± 16.8 for OWLS-II OE.

**Table 2:** Baseline Disease Characteristics - FXS Participants (Enrolled Analysis Set)

	FXS Participant (N=10)
Genetic confirmation of FXS	
Yes, n (%)	10 (100.0%)
Mosaic positive	
No, n (%)	6 (60.0%)
Yes, n (%)	4 (40.0%)
Tissue or fluid tested	
Blood, n (%)	10 (100.0%)
OWLS-II Listening Comprehension standard score	
Mean (SD)	82.0 (16.69)
Median	84.0
OWLS-II Oral Expression standard score	
Mean (SD)	73.0 (16.78)
Median	78.0
GARS-3 Social Communication scaled score	
Mean (SD)	6.7 (3.06)
Median	7.0
GARS-3 Emotional Responses scaled score	
Mean (SD)	7.7 (2.95)
Median	7.0
GARS-3 Restricted/Repetitive Behaviors scaled score	
Mean (SD)	7.7 (3.13)
Median	7.0
GARS-3 Cognitive Style scaled score	
Mean (SD)	8.0 (2.18)*
Median	8.0
GARS-3 Social Interaction scaled score	
Mean (SD)	5.3 (2.26)
Median	4.5
GARS-3 Maladaptive Speech scaled score	
Mean (SD)	7.8 (2.05)*
Median	8.0

Percentages are calculated as n/N*100. The number of subjects enrolled is used as N for all percentage calculations

*N=9

### Repeated sampling of hair follicles in FXS and healthy participants and measurement of FMRP and FMR1 mRNA

A similar number of hair follicles were obtained using repeated collection at the home and office locations in both the FXS and healthy participant groups. The majority of the hair follicles obtained from FXS participants were above the lower limit of quantification (LLOQ) for FMRP using MSD ELISA (office: 81.3% ± 19.65; home: 59.3% ± 30.35). Similar percentages were seen for the healthy participants in the study (office: 64.1% ± 23.77; home 70.0% ± 20.92). Hair follicles were collected from 15 individuals. All follicles from one individual were used to optimize assay development and were not available for measurement of FMRP. For the other 14 individuals, a total of 146 hair follicles were collected, with a mean of 10.4 follicles per person. Of the 146 hair follicles collected and measured, 100 (68.5%) had measurable FMRP protein. In fact, all 14 individuals had at least one follicle with measurable FMRP (range 4 to 11 hair follicles).

Measurement of FMRP and *FMR1* mRNA levels by any method did not show any notable variation by collection location at home versus office across the various sample collection methodologies (see Tables 3 and 4). While FMRP levels were not quantifiable for any buccal swab samples collected at either location, no variation in *FMR1* mRNA measured by quantitative reverse transcription polymerase chain reaction (qRT-PCR) was seen for buccal swab samples collected at office versus home locations (*n* = 7) in the FXS population. Values from qRT-PCR and PrimeFlow™ are not directly comparable. For healthy participants, no variation in FMRP or *FMR1* mRNA levels was seen for samples collected at office versus home locations. FMRP levels measured by MSD ELISA were similar in hair follicles versus peripheral blood lymphocytes within each group of participants.

**Table 3:** FMRP Levels by Collection Type and Collection Location – Descriptive Statistics

Reported Value									
Group	Collection Type	Collection Location	n	n BLQ	Mean	SD	Median	Min	Max
FXS Participant (N=9)	Hair Follicle	Clinic	9	1	19.235	23.4541	10.195	0.000	63.727
		Home	8	1	21.213	24.3434	11.320	0.000	64.088
	Blood	Clinic	8	2	31.964	34.0492	18.526	0.000	79.699
		Home	2	1	4.022	5.6885	4.022	0.000	8.045
Healthy Participant (N=5)	Hair Follicle	Clinic	5	0	114.520	21.1575	114.056	84.045	143.772
		Home	5	0	109.349	29.1144	109.821	81.143	155.361
	Blood	Clinic	5	0	103.252	18.0375	103.487	83.047	128.409
		Home	4	0	106.317	24.0206	108.898	79.329	128.144

*Notes:* For the hair follicle type, the mean fmol FMRP/ug protein value across all samples for each subject is summarized

For the buccal swab collection type, the fmol FMRP/ug protein value is not summarized as the value was ‘Below LLOQ’ for all samples

*Abbreviations: Max* maximum, *Min* minimum, *n* number of subjects, *SD* standard deviation, *BLQ* below limit of quantification

**Table 4:** *FMR1 mRNA* Levels by Collection Type and Collection Location – Descriptive Statistics

Reported Value									
Group	Collection Type	Collection Location	n	n BLQ	Mean	SD	Median	Min	Max
FXS Participant (N=9)	Buccal Swab	Clinic	7	1	0.354	0.3782	0.363	0.000	1.064
		Home	7	1	0.510	0.5799	0.247	0.000	1.471
	Hair Follicle	Clinic	9	0	0.090	0.1132	0.045	0.000	0.288
		Home	7	1	0.077	0.0904	0.040	0.000	0.219
	Blood	Clinic	8	0	1.012	0.1052	0.990	0.811	1.148
		Home	2	0	0.897	0.0957	0.897	0.830	0.965
Healthy Participant (N=5)	Buccal Swab	Clinic	5	0	1.108	0.8453	0.784	0.523	2.341
		Home	5	0	0.772	0.3437	0.733	0.394	1.228
	Hair Follicle	Clinic	5	1	0.165	0.1504	0.195	0.000	0.370
		Home	5	0	0.184	0.1407	0.158	0.077	0.423
	Blood	Clinic	4	0	1.197	0.0842	1.181	1.124	1.303
		Home	4	0	1.164	0.0604	1.152	1.108	1.245

*Notes:* For the hair follicle and buccal swab collection types, the mean FMR1 mRNA Delta Cq value across all samples for each subject is summarized

For the blood collection type, two Relative FMR1 values were reported for Subjects 101-002, 101-005, and 101-007; only the smallest value for each subject is summarized

Buccal and hair follicle FMR1 mRNA were quantified by qRT-PCR and blood FMR1 was measured by PrimeFlow^TM^. The 2 measurements cannot be directly compared across sample types. Within sample type comparisons between healthy participants and FXS participants are appropriate

For FMR1 PrimeFlow^TM^ values are reported as FMRP/IC (dapB) Median Fluorescent Intensity (MFI)

*Abbreviations*: *BLQ* below limit of quantification, *Max* maximum, *Min* minimum, *n* number of subjects, *SD* standard deviation

In FXS participants, mean FMRP levels at visit 1 and visit 2 were 20.7 ± 23.0 fmol/μg protein and 18.0 ± 23.7 fmol/μg protein, respectively, in hair follicles compared to 27.3 ± 31.3 fmol/μg protein and 24.1 ± 41.8 fmol/μg protein at visit 1 and visit 2, respectively, in peripheral blood lymphocytes. FMRP values for healthy participants also did not show any notable difference between the hair follicle (114.5 ± 21.1 for visit 1, 109.3 ± 29.1 for visit 2) and blood lymphocyte (103.2 ± 18.0 for visit 1 and 106.3 ± 24.0 for visit 2) by MSD.

### Repeated sampling, FMRP, and FMR1 mRNA measurement from hair follicles, peripheral blood, and buccal swabs

In FXS participants, both the hair follicle and blood lymphocyte cells showed values below the LLOQ; for analysis purposes, these values were considered to be null (i.e., zero). One null value in one hair follicle was seen for FMRP in one healthy participant. The point of failure for any lysate used for MSD was a total protein concentration below the LLOQ of the total protein assay. Only one FXS participant had no quantifiable fmol FMRP/ug protein samples (i.e., 4 failed samples and 1 below LLOQ sample). Hair follicles were collected from 15 individuals. All follicles from one individual were used to optimize assay development and are not available for measurement of FMRP. For the other 14 individuals, a total of 146 hair follicles were collected, with a mean of 10.4 follicles per person. Of the 146 hair follicles collected and measured, 100 (68.5%) had measurable FMRP protein. In fact, all 14 individuals had at least one follicle with measurable FMRP (range 4 to 11 hair follicles).

Both blood lymphocyte and hair follicle FMRP levels measured by MSD were lower by a factor of 4 to 6 in FXS participants compared to healthy participants. In all subjects, FMRP could not be detected in buccal mucosa sampled for FMRP determination in this study. Of the 2 subjects who were negative for FMRP using blood samples, one of them also was negative for FMRP in the hair follicle sample.

FMRP was quantified in hair follicles using only the MSD ELISA. FMRP was quantified in PBMCs using two methods. An absolute quantity in fmol/μg total protein was determined using the MSD ELISA; and a relative quantification in a ratio of FMRP Mean Fluorescence Intensity to Isotype Control Mean Fluorescence Intensity using the multiparameter, flow cytometric assay PrimeFlow™.

**Fig. 1:**
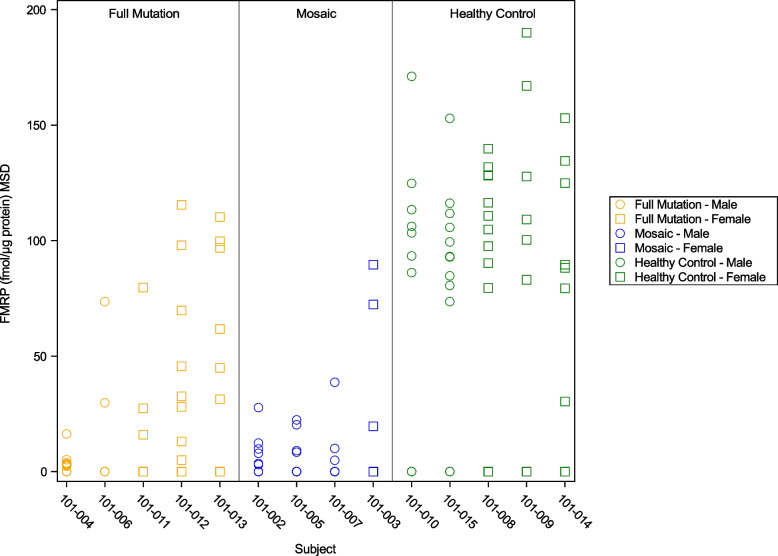
FMRP level per sampled hair follicle by subject clustered by group and sex

Per follicle, FMRP levels detected using MSD showed clustering by FXS diagnosis with higher FMRP levels seen in healthy controls compared to subjects with the full mutation or mosaic subjects (Fig. 1). Inter-follicle variability was lowest in mosaic subjects with the majority of follicles showing FMRP levels less than 25 fmol/μg protein.

**Fig. 2:**
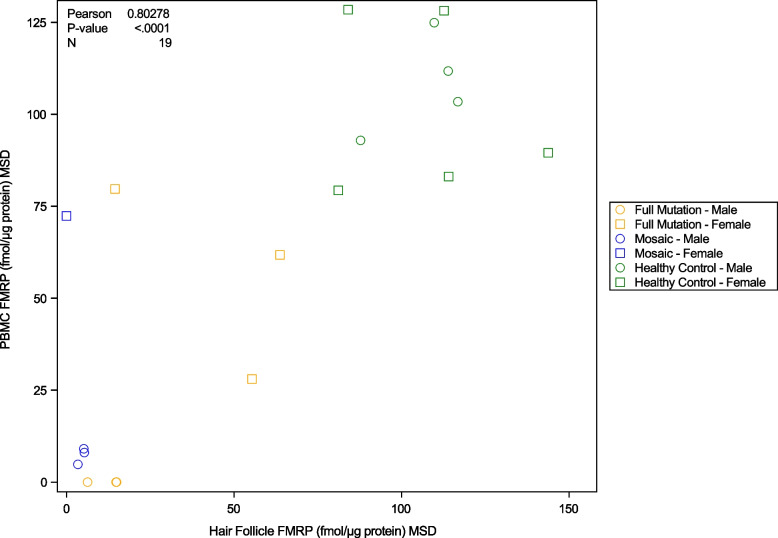
Scatterplot of mean FMRP level across sampled hair follicles and PBMC FMRP level clustered by group and sex

Mean FMRP in hair follicles was related to that in PBMCs (Pearson’s *r* = 0.803; *p* < 0.0001) with full mutation status subjects and healthy controls showing higher levels of FMRP than mosaic subjects when assessed using MSD (Fig. 2). Full mutation subjects showed intermediate FMRP levels compared to healthy controls and mosaic subjects; 4 full mutation subjects, however, showed low FMRP levels with the hair follicle method of which 2 subjects showed intermediate levels and 2 subjects showed corroborating low levels with the PBMCs (overall, *n* = 6). The relationship in PBMCs between the PrimeFlow™ and MSD ELISA methods showed a similar pattern of high levels of FMRP in healthy control (male/female subjects), intermediate levels of FMRP in full mutation female subjects, and low levels in full mutation male subjects and in mosaic (male/female) subjects, respectively.

Hair follicle mean *FMR1* mRNA levels were lower in FXS participants compared to healthy participants (Table 5). The mean hair follicle *FMR1* mRNA level using qRT-PCR in FXS participants was 0.055 ± 0.10 Delta Cq and 0.127 ± 0.11 Delta Cq compared to 0.165 ± 0.15 Delta Cq and 0.184 ± 0.14 Delta Cq for healthy participants at visit 1 and visit 2, respectively. Mean *FMR1* mRNA in blood lymphocytes assessed using PrimeFlow™ showed less variation in relative terms at 1.026 ± 0.08 *FMR1* mRNA/dapB MFI and 0.905 ± 0.15 *FMR1* mRNA/dapB MFI in FXS participants and 1.197 ± 0.08 *FMR1* mRNA/dapB MFI and 1.164 ± 0.06 *FMR1* mRNA/dapB MFI in healthy participants at visit 1 and visit 2, respectively, compared to hair follicle *FMR1* levels using qRT-PCR. *FMR1* mRNA was not quantified in PBMCs using qRT-PCR. Mean *FMR1* mRNA assessed by qRT-PCR using the buccal swab showed a mean level of 0.354 ± 0.38 Delta Cq and 0.443 ± 0.54 Delta Cq in FXS participants compared to 1.108 ± 0.85 Delta Cq and 0.772 ± 0.34 Delta Cq for healthy participants at visit 1 and visit 2, respectively. Higher *FMR1* mRNA levels were generally seen in healthy controls and mosaic subjects, although there were a number of healthy control and mosaic follicles with intermediate and low *FMR1* mRNA levels. The majority of full mutation subjects demonstrated low *FMR1* mRNA levels.

**Table 5:** *FMR1 mRNA* Levels and Changes from Baseline by Collection Type and Visit

		Reported Value						Change from Baseline Value^a^					
Collection Type	Visit	n	Mean	SD	Median	Min	Max	n	Mean	SD	Median	Min	Max
**FXS Participant (N=9)**													
Buccal Swab	Visit 1	8	0.354	0.3782	0.363	0.000	1.064						
	Visit 2	7	0.443	0.5438	0.235	0.000	1.471	5	0.138	0.7450	0.056	-0.840	1.108
Hair Follicle	Visit 1	8	0.055	0.0952	0.030	0.000	0.288						
	Visit 2	9	0.127	0.1055	0.078	0.000	0.228	8	0.082	0.1665	0.111	-0.247	0.271
Blood	Visit 1	7	1.026	0.0750	0.984	0.965	1.148						
	Visit 2	3	0.905	0.1476	0.830	0.811	1.075						
**Healthy Participant (N=5)**													
Buccal Swab	Visit 1	5	1.108	0.8453	0.784	0.523	2.341						
	Visit 2	5	0.772	0.3437	0.733	0.394	1.228	4	-0.336	0.8542	-0.004	-1.595	0.258
Hair Follicle	Visit 1	5	0.165	0.1504	0.195	0.000	0.370						
	Visit 2	5	0.184	0.1407	0.158	0.077	0.423	5	0.019	0.1874	0.077	-0.212	0.228
Blood	Visit 1	4	1.197	0.0842	1.181	1.124	1.303						
	Visit 2	4	1.164	0.0604	1.152	1.108	1.245	1	-0.119		-0.119	-0.119	-0.119

*Notes:* For the hair follicle and buccal swab collection types, the mean FMR1 mRNA Delta Cq value across all samples for each subject is summarized

For the blood collection type, two Relative FMR1 values were reported for Subjects 101-002, 101-005, and 101-007; only the smallest value for each subject is summarized

*Abbreviations: Max* maximum, *Min* minimum, *n* number of subjects, *SD* standard deviation

^a^Baseline is defined as value collected during Visit 1. Change from baseline values are calculated as the assessment value minus the baseline value

In hair follicles, mean FMRP by MSD ELISA did not correlate with mean *FMR1* mRNA by qRT-PCR in healthy controls compared to mosaic and full mutation subjects, and full mutation subjects also showed no correlation compared to mosaic subjects. All mosaic subjects (*n* = 4) showed low levels of FMRP expression. In PBMCs, mean FMRP positively correlated with mean *FMR1* mRNA in both healthy controls and full mutation participants. PrimeFlow™ (a single-cell method) was used to measure both FMRP and *FMR1* mRNA simultaneously in PBMCs. By PrimeFlow™, PBMCs from mosaic participants gave 2 distinct *FMR1* mRNA populations; therefore, results from mosaic participants were not included.

### Associations of FMRP and FMR1 mRNA with measures of language and behavior (OWLS-II LC, OWLS-II OE, and GARS-3)

A positive Pearson’s r correlation was seen between *FMR1* mRNA levels and the OWLS-II LC and OE subscale scores in FXS subjects. The highest correlation between *FMR1* mRNA levels and OWLS-II LC and OE scores was seen for the FXS blood samples (0.668 and 0.666, respectively), followed by samples collected by buccal swab (0.444 and 0.595, respectively) and hair follicle plucking (−0.189 and −0.286, respectively) (see Tables 6 and 7). This data suggests that measurements in blood better reflect the clinical phenotype than measurements in buccal swabs or hair follicles. For the GARS-3, negative correlations were seen with the *FMR1* mRNA levels from blood samples and the Restricted/Repetitive Behavior and Maladaptive Speech subscale scores (−0.555 and −0.702, respectively); other GARS-3 subscale scores showed correlations with blood *FMR1* mRNA levels ranging from −0.084 to −0.234.

**Table 6:** Relationship between *FMR1 mRNA* levels and OWLS-II score at visit 1 — Pearson correlation coefficients

OWLS-II Listening Comprehension subscale					OWLS-II Oral Expression subscale	
Group	Collection type	*n*	Pearson’s r	*p*-value	Pearson’s r	*p*-value
FXS participant (*N* = 9)	Buccal swab	7	0.444	0.319	0.595	0.159
	Hair follicle	8	−0.189	0.654	−0.286	0.493
	Blood	7	0.668	0.101	0.666	0.102
Healthy participant (*N* = 5)	Buccal swab	4	−0.709	0.291	0.210	0.790
	Hair follicle	5	−0.117	0.851	−0.256	0.678
	Blood	4	0.987	0.013	0.015	0.985

**Table 7:** Relationship between *FMR1 mRNA* levels and GARS-3 score at visit 1 — Pearson correlation coefficients

			GARS-3 Emotional Response		GARS-3 Social Communication		GARS-3 Restricted/Repetitive Behavior		GARS-3 Social Interaction		GARS-3 Cognitive Style		GARS-3 Maladaptive Speech	
Collection														
Group	type	*n*	Pearson’s r	*p*-value	Pearson’s r	*p*-value	Pearson’s r	*p*-value	Pearson’s r	*p*-value	Pearson’s r	*p*-value	Pearson’s r	*p*-value
FXS participant (*N* = 9)	Buccal swab	7	−0.343	0.452	0.15496	0.740	−0.335	0.463	−0.024	0.959	0.133	0.802	−0.436	0.388
	Hair follicle	8	−0.227	0.589	0.26244	0.530	0.518	0.188	−0.125	0.768	0.542	0.209	0.642	0.120
	Blood	7	−0.207	0.656	−0.19646	0.673	−0.555	0.196	−0.084	0.857	−0.234	0.613	−0.702	0.079
Healthy participant (*N* = 5)	Buccal swab	4	nc		0.97289	0.027	nc		nc		0.890	0.110		nc
	Hair follicle	5	0.763	0.133	−0.48042	0.413	nc		nc		−0.120	0.848		nc
	Blood	4	−0.492	0.508			nc		nc		0.169	0.831		nc

*Notes:* For the hair follicle and buccal swab collection types, the mean FMR1 mRNA Delta Cq value across all samples is correlated with each OWLS-II score or each GARS-3 score. For the blood collection type, two relative FMR1 values were reported for subjects 101-002, 101-005, and 101-007; only the smallest value for each subject is used in correlations

*Abbreviations: GARS-3* Gilliam Autism Rating Scale 3rd Edition, *nc* not calculable *OWLS-II* Oral and Written Language Scale 2nd Edition

FMRP levels also showed a high level of positive correlation with the OWLS-II LC and OE subscale scores in FXS subjects, with relatively higher correlation seen for blood samples compared to hair follicle samples (see Tables 8 and 9). In blood, Pearson’s *r* statistics were 0.774 and 0.777 between FMRP levels and the OWLS-II LC and OE subscale, respectively. For the GARS-3, negative correlations were seen between FMRP levels and the Restricted/Repetitive Behavior and Maladaptive Speech subscale scores with both blood and hair follicle samples in FXS participants.

**Table 8:** Relationship between FMRP levels and OWLS-II score at visit 1 — Pearson correlation coefficients

		OWLS-II Listening Comprehension subscale			OWLS-II Oral Expression subscale	
Group	Collection type	*n*	Pearson’s r	*p*-value	Pearson’s r	*p*-value
FXS participant (*N* = 9)	Hair follicle	9	0.384	0.307	0.619	0.076
	Blood	7	0.774	0.041	0.777	0.040
Healthy participant (*N* = 5)	Hair follicle	5	−0.430	0.470	0.596	0.288
	Blood	5	−0.071	0.910	−0.731	0.160

**Table 9:** Relationship between FMRP levels and GARS-3 score at visit 1 — Pearson correlation coefficients

			GARS-3 Emotional Response		GARS-3 Social Communication		GARS-3 Restricted/Repetitive Behavior		GARS-3 Social Interaction		GARS-3 Cognitive Style		GARS-3 Maladaptive Speech	
Collection														
Group	Type	n	Pearson's r	*P-*value	Pearson's r	*P-*value	Pearson's r	*P-*value	Pearson's r	*P-*value	Pearson's r	*P-*value	Pearson's r	*P-*value
FXS Participant (N=9)	Hair Follicle	9	-0.302	0.429	0.008	0.984	-0.608	0.082	0.276	0.473	-0.204	0.629	-0.561	0.148
	Blood	7	-0.203	0.663	-0.187	0.688	-0.650	0.114	0.060	0.899	-0.370	0.414	-0.790	0.035
Healthy Participant (N=5)	Hair Follicle	5	-0.017	0.978	0.062	0.921	nc		nc		-0.577	0.308		nc
	Blood	5	0.264	0.668	0.007	0.991	nc		nc		0.582	0.304		nc

*Notes:* For the hair follicle collection type, the mean fmol FMRP/μg protein value across all samples is correlated with each OWLS-II score or each GARS-3 score. For the buccal swab collection type, the fmol FMRP/μg protein value is not summarized as the value was “below LLOQ” for all samples

*Abbreviations: GARS-3* Gilliam Autism Rating Scale 3rd Edition, *nc* not calculable, *OWLS-II* Oral and Written Language Scale 2nd Edition

## Discussion

This was a single-center, prospective, nondrug pilot biomarker feasibility study. We assessed repeated collection of hair follicles by plucking in individuals with FXS and those who were typically developing for the quantitative measurement of FMRP and *FMR1* mRNA. Results were compared to blood and buccal swabs and collected at two different locations, in their home and in the clinic. The primary objective of this study was to determine whether repeated collection of scalp hair follicles by plucking is feasible in individuals with FXS. Secondary objectives included comparison of the completeness of data in office visits versus home visits, comparisons of the positivity and levels of FMRP and *FMR1* mRNA in hair follicles by collection type and collection location, and assessment of associations between FMRP and *FMR1* mRNA obtained by above methods with measures of clinical severity. Given that measurement of FMRP by IHC was discontinued early due to fragility of youth hair follicles, one of the initial goals of the study, to measure if the presence and amount of FMRP by MSD ELISA were less variable and more reliable than with IHC, was not done.

Mostly young males and female subjects were enrolled into the study with all FXS subjects confirmed with the full mutation, almost half of which were mosaic positive. For full FXS subjects, the mean OWLS-II LC score was 82 ± 16.7 (range 54 to 108), and the mean OWLS-II OE score was 73 ± 16.8 (range 48 to 95). Scores below 70 are considered deficient (OWLS-II: average = 85–115; below average = 70–84; deficient = less than 70).

This study determined that the repeated collection of scalp follicles by plucking was a feasible method of collection in children with FXS. For the FXS group, a mean of 4.7 ± 1.00 hair follicles per subject were collected at the office visit (*n* = 9) versus a mean of 6.1 ± 2.23 hair follicles per subject at the home visit (*n* = 8). This was similar to the collection in healthy participants with a mean of 5.2 ± 2.17 hair follicles collected per subject at the office visit (*n* = 5) compared to a mean of 4.8 ± 1.79 hair follicles per subject at the home visit (*n* = 5). Based on our hair follicle collection and analyses, we believe an average of 10 hair follicles collected per person is adequate for collection in future studies.

Using the newly developed method of MSD ELISA, similar mean percentages of FMRP-positive hair follicles obtained using plucking were seen from FXS and healthy participants (FXS participants — office: 81.3%, home: 59.3%; healthy participants — office: 64.1%, home: 70.0%). Absolute levels of FMRP and *FMR1* mRNA were substantially higher in healthy participants compared to full mutation and mosaic FXS participants and lowest in the FXS boys. Importantly, measurement of FMRP and *FMR1* mRNA levels did not show any notable variation by collection location at home versus the office across the various tested sample collection methodologies of hair follicle, blood sample, and buccal swab.

In hair follicles, we observed no relationship between FMRP measured by MSD and *FMR1* mRNA measured by qRT-PCR. These findings are similar to those noted in Schneider et al. [32], where there was no clear relationship between *FMR1* mRNA expression and FMRP protein levels. However, in PBMCs using the novel PrimeFlow™ assay, we did observe a strong relationship between the relative amounts of FMRP and *FMR1* mRNA. Further studies may be warranted to determine if the lack of relationship in hair follicles for FMRP and *FMR1* mRNA is due to the use of divergent techniques or if it is a property of hair follicles or the collection methods. In general, there was little variation in FMRP and *FMR1* mRNA levels between successive visits indicating a favorable repeatability profile for hair follicle sampling, an important characteristic for potential use of one or more of these biomarkers in the context of future therapeutic clinical trials that seek to reactivate *FMR1* mRNA*/*FMRP. Notably, hair follicle values demonstrated that if a sufficient number of samples are obtained from FXS participants, FMRP is not only detectable but also quantifiable in full mutation participants. Use of blood lymphocytes allow for only one sampling per visit. The sampling superiority of hair follicles versus blood lymphocytes may allow for the quantification of FMRP by MSD in full mutation individuals and allows a determination of percent positive follicles.

Strong positive correlations were seen between FMRP and *FMR1* mRNA levels in blood and the OWLS-II LC and OE subscale scores in FXS subjects and of greater magnitude than for hair follicles and buccal swabs. This confirms our previous recent finding in a separate population of children with FXS [5]. For blood samples, Pearson’s correlations were high at 0.668 and 0.666 between FMRP levels and the OWLS-II LC (*p* = 0.101) and OE subscales (*p* = 0.102), respectively. Similar correlations were observed between *FMR1* mRNA and GARS-3 Restricted/Repetitive Behavior and Maladaptive Speech subscales (*p* = 0.114 and *p* = 0.035, respectively). Thus, the study provides strong support for the use of blood samples to measure longitudinal changes in FMRP/*FMR1* mRNA in future therapeutic clinical trials seeking to treat the root cause of FXS. The strong correlations of blood with clinical outcome assessments of communication such as OWLS-II support the clinical significance of this biomarker. Moreover, above findings are similar to those demonstrated in Roth et al. (2021), in which a positive association between IQ and FMRP expression in PBMCs by either MSD or PrimeFlow™ was noted. However, several studies have demonstrated that venipuncture in children with ASD and other neurodevelopmental disorders can lead to significant distress, behavioral disturbance, noncompliance with study procedures, and withdrawal from the study [24, 25, 33, 34]. These findings were corroborated in the current study as well in which we noted that blood samples were only obtained from 6 out of 10 FXS participants. As described in Berry-Kravis et al. (2013) [35] and the NIH Outcome Measures Working Groups, use of focused assessments of core cognitive features is recommended for outcome measurements in clinical trials, and hence, measures of language, social communication, and behaviors were chosen for our study. The lack of findings in the FXS group could be explained by the small sample size of our study, or the relationship may not apply across the entire range of GARS-3 scores, perhaps due to a floor effect of the measure. Also, there is limited data on the GARS-3 in the FXS population [36].

### Future directions

Future work replicating these findings with a larger sample size may improve the feasibility of repeated collection of blood in children with FXS. As described above, upon establishment of the feasibility of these methods, the next crucial step would involve testing of potential treatments for the root cause of FXS and subsequent development of translational methods to measure reactivation of *FMR1* mRNA and FMRP in proof of concept (POC).

### Limitations

This was a single-center study with a small sample size and a relatively high proportion of mosaics. There were issues with sample heterogeneity with 4/10 FXS participants being female. Technical issues were noted with hair follicles such as unreliable protein concentration measurements. Lysing a single hair follicle in 50 μL of RIPA (minimum volume) results in a lysate not suitable for BCA assay. The lower limit of detection for the BCA assay is 20 μg/mL, and it requires 25 μL of sample. The single follicle lysates are not suitable for the BCA assay because (1) the majority of the single hair follicle lysate protein concentrations are < 20 μg/mL and (2) the volume requirement for the BCA assay would have consumed most of the sample leaving not enough to test in the MSD assay. We needed about 15 follicles in 50 μL RIPA to get a concentration sufficient to be detected by BCA. Due to these technical challenges, we developed our capillary electrophoresis assay for total protein determination. Using the capillary electrophoresis total protein method, the total protein in a single hair follicle ranged from 1.7 to 202 ug/mL. This wide range is most likely due to two factors: (1) which of the 3 growth phases the follicle was in anagen, catagen, or telogen and (2) if the pluck resulted in removal of a complete follicle or a partial follicle. Additionally, a high variability in FMRP levels was noted, regardless of the biosample source. The lack of difference between sample sources is most likely a reflection of this variability despite the higher mean values of blood samples. Only nominal *p*-values were calculated for the various correlational analyses; no correction for multiplicity was applied. As noted above, there is a paucity of existing literature examining psychometric properties and use of the GARS-3 in the FXS population as an outcome measure for clinical trials.

## Conclusion

In summary, our data support the feasibility of repeated sampling of hair follicles in individuals with FXS, in both home and office settings, for measurement of FMRP and FMR1 mRNA levels. While FMRP and *FMR1* mRNA obtained from hair follicles did not demonstrate strong relationships with language and behavioral measures, little variation in their levels during successive visits was noted, which is an important feature in biomarker development to plan future studies. Furthermore, the current study provides support for the use of repeated hair follicle sampling in future clinical therapeutic studies as an effective biomarker of FMRP and *FMR1* mRNA levels and highlights the need to improve the feasibility of repeated collection of blood in these individuals.

## Supplementary Information

**Additional file 1: Supplemental Table 1.** Study Participant Characteristics
