# Supplementary material for: The feasibility and utility of hair follicle sampling to measure FMRP and FMR1 mRNA in children with or without fragile X syndrome: a pilot study
Source: J Neurodev Disord. 2022 Dec 9;14:57. doi: 10.1186/s11689-022-09465-7 (PMC9733195; doi:10.1186/s11689-022-09465-7)
Supplement: Supplementary file 1 — Additional file 1: Supplemental Table 1. Study Participant Characteristics [file 11689_2022_9465_MOESM1_ESM.docx]

**Supplemental Table 1:**

**Study Participant Characteristics:**

|  |  |  |  |  | **Hair** | **Follicle** | **Buccal** | **Swab** | **PBMC** |  |
| --- | --- | --- | --- | --- | --- | --- | --- | --- | --- | --- |
| **Case** | **Age (years)** | **Sex** | **Category** | **CGG Repeat** | **MSD FMRP fmol** | ***FMR1* mRNA (qPCR)** | **MSD FMRP fmol** | ***FMR1* mRNA (qPCR)** | **MSD FMRP fmol** | ***FMR1* mRNA (PrimeFlow^TM^) FMR1 MFI / dapB MFI** |
| **1** | 4 | M | Mosaic | >200, 84 (±3) | Not available* | Not available* | Not available* | Not available* | Not available* | Not available* |
| **2** | 5 | M | Mosaic | >200, >200 | 0.8319 | 0.0059 | Below LLOQ | 0.000 | 1.1754 | 0.9651/3.6345 |
| **3** | 3 | M | Full mutation | >200 | 1.0026 | 0.0414 | Below LLOQ | Failed | 12.6930 | 0.8106 |
| **4** | 5 | F | Full mutation | >200 | 0.6482 | 0.0009 | Below LLOQ | 0.000 | Below LLOQ | 1.0752 |
| **5** | 7 | M | Mosaic | 120, 200 and >200 | 0.7778 | 1.1003 | Below LLOQ | 0.4448 | 1.6712 | 0.9964/3.2580 |
| **6** | 10 | M | Full mutation | >200 | 0.7047 | 0.0002 | Below LLOQ | 0.0226 | Below LLOQ | 0.9837 |
| **7** | 5 | M | Mosaic | 100, 260, 500 | 0.8898 | 0.0199 | Below LLOQ | 0.0574 | 1.3549 | 0.9823/3.5885 |
| **8** | 4 | F | Healthy control | Not available | 2.4046 | 0.4727 | Below LLOQ | 1.1069 | 34.1714 | 1.2271 |
| **9** | 9 | M | Healthy control | Not available | 12.2564 | 0.0000 | Below LLOQ | 0.6854 | 31.5323 | 1.3031 |
| **10** | 11 | F | Healthy control | Not available | 21.9830 | 0.1183 | Below LLOQ | 2.2635 | 32,5416 | 1.2448 |
| **11** | 7 | F | Full mutation | >200 | 0.6482 | 0.0964 | Below LLOQ | 0.3485 | 19.4997 | 1.1477 |
| **12** | 9 | F | Full mutation | >200 | 9.4696 | 0.0304 | Below LLOQ | 0.4805 | 9.9449 | 0.9838 |
| **13** | 13 | F | Full mutation | 430-870 | 5.8320 | 0.0045 | Below LLOQ | 1.0569 | 16.0788 | 1.1203 |
| **14** | 7 | F | Healthy control | Not available | 8.0800 | 0.4615 | Below LLOQ | 0.5076 | 33.9144 | 1.1241 |
| **15** | 9 | M | Healthy control | Not available | 2.7964 | 0.2455 | Below LLOQ | Failed | 28.3976 | 1.1353 |

* Samples that were collected from Participant 1 were used to pressure test the MSD assay system. Since the total protein would be below the LLOD of the BCA assay, an ELISA to quantify beta actin was used to normalize samples. However, this method it did not work in the samples collected from this cohort of children that was discovered after running collections from Participant 1. Hence, we developed the capillary electrophoresis (ProteinSimple) method to determine total protein. Unfortunately, not enough material was left from this participant to run in the redesigned workflow.

Abbreviations: LLOQ: Lower limit of quantification; MSD: mesoscale discovery
